# Nuclear morphogenesis: forming a heterogeneous nucleus during embryogenesis

**DOI:** 10.1242/dev.200266

**Published:** 2022-02-17

**Authors:** Albert Tsai, Justin Crocker

**Affiliations:** European Molecular Biology Laboratory, 69117 Heidelberg, Germany

**Keywords:** Biophysics, *Drosophila melanogaster*, Nuclear organization, Super-resolution fluorescence imaging

## Abstract

An embryo experiences increasingly complex spatial and temporal patterns of gene expression as it matures, guiding the morphogenesis of its body. Using super-resolution fluorescence microscopy in *Drosophila melanogaster* embryos, we observed that the nuclear distributions of transcription factors and histone modifications undergo a similar transformation of increasing heterogeneity. This spatial partitioning of the nucleus could lead to distinct local regulatory environments in space and time that are tuned for specific genes. Accordingly, transcription sites driven by different *cis*-regulatory regions each had their own temporally and spatially varying local histone environments, which could facilitate the finer spatial and temporal regulation of genes to consistently differentiate cells into organs and tissues. Thus, ‘nuclear morphogenesis’ may be a microscopic counterpart of the macroscopic process that shapes the animal body.

## Abstract (precis)

**Summary:** The nuclear space becomes progressively heterogeneous during embryo development, forming distinct transcriptional sub-nuclear environments, paralleling the partitioning of the embryo body during morphogenesis.

## INTRODUCTION

During embryogenesis, transcriptional regulation controls the expression of genes that pattern the animal body (Long et al., 2016; Mallo and Alonso, 2013; Reiter et al., 2017; Spitz and Furlong, 2012). This process involves interactions between transcription factors (TFs), chromatin regulators and genome topology. Genome-wide assays have revealed a diversity of regulatory elements and the factors that bind them (ENCODE Project Consortium, 2012; Johnson et al., 2007; Kundaje et al., 2015; Stunnenberg et al., 2016; Thurman et al., 2012). Notably, these assays have detected reorganization within the nucleus during cellular differentiation, where longer range interactions consolidate chromatin regions and genes into topologically associated domains (TADs). These observations suggest that cellular differentiation involves transforming the nucleus from a uniform compartment into a heterogeneous and partitioned space. However, these maps represent averages over populations of cells, and therefore mask cellular and regulatory heterogeneity. There is now recognition that cell-to-cell variation of TFs, chromatin modifications and interactions between regulatory elements are crucial for animal development (Furlong and Levine, 2018; Shema et al., 2019; Tanay and Regev, 2017). However, a challenge in the field has been to pair single-cell and genomics techniques with complex tissues and developmental systems.

Improvements in imaging technology are advancing the capability to measure regulatory interactions in developmental systems at the single cell and locus level (Chen et al., 2018; Garcia et al., 2013; Lim et al., 2018; Mir et al., 2017, 2018; Tsai et al., 2017). These techniques can spatially trace genome structures at high resolution (Bintu et al., 2018; Mateo et al., 2019; Shema et al., 2019), allowing direct observations of the transcriptional environments around individual gene loci in specific cells. We have previously found that the distributions of TFs were heterogeneous in the nuclei of *Drosophila* embryos at a late stage of development and that the developmental gene *shavenbaby* (*svb*; *ovo* – FlyBase) resided in regions enriched for TFs when it is transcriptionally active (Tsai et al., 2017).

However, the origins and spatial-temporal behaviors of these transcriptional microenvironments during development remain unclear. When does the nuclear microenvironment form? How and when do genes interact with these microenvironments? Here, we imaged the distributions of TFs and histone modifications in *Drosophila melanogaster* embryos across development. Additionally, we imaged histone modifications at transcription sites driven by *hunchback* (*hb*), *rhomboid* (*rho*) and *snail* (*sna*) enhancers to measure their local chromatin environment during different stages of development and at different locations in the embryo. In summary, we found that the distributions of TFs and epigenetic marks became progressively heterogeneous as development proceeded. Thus, the nuclear space may undergo a transformation that parallels morphogenesis, where the animal body becomes increasingly heterogeneous and segmented. Additionally, even for the same gene, the nuclear environments at transcription sites depended on the location and timing of expression. Organizing the nucleus to form multiple reconfigurable regulatory environments in a single nucleus could permit genes to perform different tasks at different times and facilitate the complicated regulatory patterns during later stages of embryogenesis.

## RESULTS AND DISCUSSION

### Increasing heterogeneity of TF distributions as development progresses

**Fig. 1.**
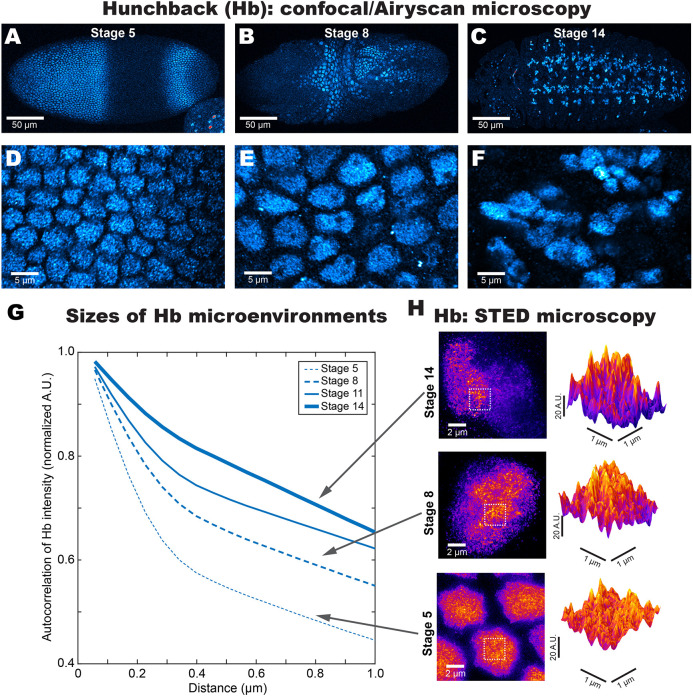
**Increasing heterogeneity of Hunchback (Hb) distribution as embryo development progresses.** (A-C) The distribution of Hb in *Drosophila* embryos from (A) stage 5, (B) stage 8 and (C) stage 14. (D-F) A detailed view of the nuclear distribution of Hb using Airyscan from the panels above. (G) The radially averaged autocorrelation function of Hb. See Fig. S1A-C for how to interpret autocorrelation plots. (H) STED microscopy of nuclei from stage 5 (bottom), stage 8 (middle) and stage 14 (top).

Hunchback (Hb) is a TF that is a gap gene in the early embryo (Nüsslein-Volhard and Wieschaus, 1980). We observed that Hb established the classical pattern at stage 5 (Fig. 1A). Hb expression moved into neuroblasts at stage 8 (Fig. 1B) and into the ventral nerve cord at stage 14 (Fig. 1C) (Kambadur et al., 1998). To investigate whether the nuclear distribution of Hb also undergoes a broad-to-specific transition during development, we stained for Hb using immunofluorescence (IF) and imaged its distributions using high-resolution confocal microscopy with Airyscan (Sheppard et al., 2013) (Fig. 1D-F).

To quantify the spatial distributions of Hb, we computed the spatial autocorrelation of Hb intensity (Fig. 1G). The level of correlation (*y*-axis) when the image has been shifted by a given distance compared with the original (*x*-axis) indicates the abundance of structures at that length scale (see Fig. S1A-C and its legends for how to interpret autocorrelation plots). There was a monotonous increase in autocorrelation as the embryo ages, suggesting that Hb environments spanning several hundred nanometers became more prevalent as development progressed. Indeed, stimulated emission depletion (STED) (Blom and Widengren, 2017) imaging showed that, at stage 5 (Fig. 1H, bottom), Hb was uniformly distributed across the nucleus. At stage 8 (Fig. 1H, middle), Hb began to coalesce into distinct nuclear regions. At stage 14 (Fig. 1H, top), this heterogeneity progressed further, with regions of high Hb concentration next to regions with almost no Hb. We also imaged Krüppel (Kr, Fig. S1D) distributions across similar developmental stages with Airyscan and computed their autocorrelation (Fig. S1E). There was a similar upward trend, suggesting the formation of local TF environments in older embryos.

The TFs Ultrabithorax (Ubx, Fig. S1F,G) and Engrailed (En, Fig. S1H-I), which are active within a narrower time frame during development, showed high autocorrelation when they are first expressed (stage 10) and when they reach maximum expression (stage 14) in nuclei on the ectoderm. At stage 16, when both are no longer expressed, their distributions became sparse with low autocorrelation. This two-state behavior for factors only expressed at later stages of development is unlike Hb and Kr, which showed a progressive increase in autocorrelation with intermediate states. This suggests that the general distributive properties of TFs may conform to the underlying nuclear architecture at the time of their expression.

### Increasing heterogeneity of histone modifications as development progresses

**Fig. 2.**
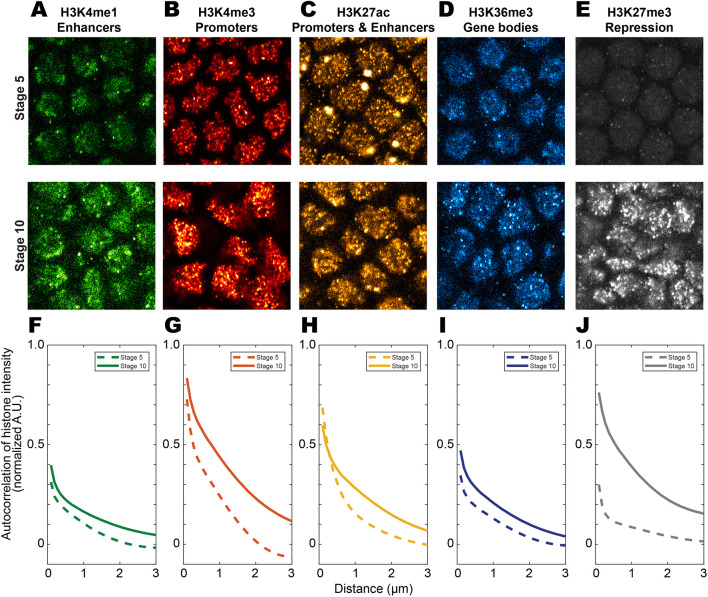
**Increasing levels of histone modification as a function of embryo age.** (A-E) The levels of histone modification of embryos at stages 5 and 10. (A) H3K4me1 (enhancers), (B) H3K4me3 (promoters), (C) H3K27ac (active enhancers and promoters), (D) H3K36me3 (active gene bodies) and (E) H3K27me3 (repressed regions) were imaged. (F-J)The autocorrelation functions of the histone modifications. All images are taken from regions with active *hbBAC* transcription sites.

Because TFs interact with DNA, we suspected that the state of the chromatin would influence where factors localize. We therefore imaged histone modifications associated with different chromatin features using high-resolution confocal microscopy to track their spatial distributions. We focused on the ventral ectodermal region where *hunchback* (*hb*) is expressed (located using *hbBAC* transcriptions sites, see the next section) and imaged embryos at stages 5 and 10, in nuclei within 20 µm of the ectoderm to preserve optical resolution. We stained for H3K4me1 (Fig. 2A, enhancers), H3K4me3 (Fig. 2B, promoters), H3K27ac (Fig. 2C, active promoters and enhancers), H3K36me3 (Fig. 2D, active gene bodies) and H3K27me3 (Fig. 2E, repressed regions) using IF.

In general, the trend was an increasing intensity of histone modifications as the embryo aged from stage 5 to 10 (Fig. 2A-E). H3K27me3 increased the most, and went from barely detectible to condensed regions with very high intensities. The autocorrelation functions of all histone modifications (Fig. 2F-J) increased across all length scales. H3K27me3 (Fig. 2J) showed the largest change followed by H3K4me3 (Fig. 2G). Overall, histone modifications became established and spatially heterogeneous in older embryos, with repressive modifications showing the strongest increase.

### Changing histone modifications around transcription sites over time

**Fig. 3.**
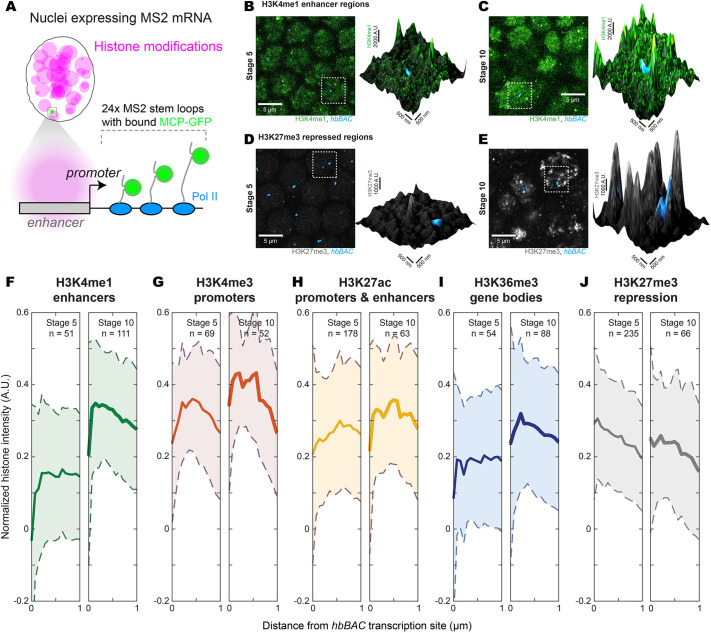
**Temporal changes in the histone environments at *hbBAC* transcription sites.** (A) Marking transcription sites of a MS2 reporter gene driven by a *cis*-regulatory region using MCP-GFP and immunofluorescence (IF) amplification preserves the signal of histone modifications. (B-E) H3K4me1 (B,C) and H3K27me3 (D,E) at stages 5 and 10, respectively. See Fig. S2 for a comparison with FISH. (F-J) The radially averaged distributions of histone modifications around *hbBAC* transcription sites. The number of transcription sites (*n*) is indicated in the figure panels. The solid line is the mean; the shaded region is the variance.

Changing histone modification patterns over time could alter the chromatin environments around genes active across multiple developmental stages, such as *hunchback* (*hb*). However, using RNA fluorescence *in situ* hybridization (FISH) to detect transcription sites degraded the signal from histone modifications (Fig. S2A,B). To improve quantitative image analysis, we detected transcription sites without the denaturing step in RNA FISH to preserve the signal from histone modifications (Fig. 3A). In brief, we crossed a *Drosophila melanogaster* line expressing a reporter mRNA containing MS2 stem loops driven by 18 kb of the *cis*-regulatory region of *hb* (*hbBAC*) (Bothma et al., 2015) with a line expressing MCP-EGFP to label the mRNA with GFP (Garcia et al., 2013). This specific cross was used for live imaging of *hb* expression kinetics in stage 5 embryos (Bothma et al., 2015). The mRNA transcription signal was then amplified using IF against GFP, in conjunction with staining for histone modifications (Fig. S2C,D for H3K4me3 and Fig. 3B-E for H3K4me1 and H3K27me3). Transcription sites for *hbBAC* appeared in a radial band towards the anterior of stage 5 embryos and in neuroblasts in stage 10 embryos, consistent with the localization of Hb in Fig. 1A,B.

To quantify the spatial distribution of histone marks around transcription sites, we computed the average radial intensity distribution centered on the transcription site (Fig. 3F-J) (according to Tsai et al., 2017). We observed a clear increase in H3K4me1 between stages 5 and 10 (Fig. 3F), while H3K4me3 (Fig. 3G) showed only a small increase and was high at both stages, which is the opposite of the global trend for these two marks (Fig. 2F,G). H3K27ac (Fig. 3H) did not change, but H3K36me3 (Fig. 3I) showed a moderate increase. Interestingly, some histone modifications showed an initial dip in intensity. The repressive mark H3K27me3 (Fig. 3J) had a different functional form, with no initial dip and a gentle downward slope. There was little change between stages 5 and 10, despite the increase in H3K27me3 throughout the nucleus going from stage 5 to 10 (Fig. 2J). In summary, the histone environment around *hbBAC* transcription sites between stages 5 and 10 showed changes that are distinct from the global trend in Fig. 2F-J.

To test whether we can resolve finer temporal changes in local histone environments, we focused on the 1 h cellularization process at end of the rapid nuclear division cycles at stage 5 (Lecuit and Wieschaus, 2000), which we divide into three phases: early, mid and late (Fig. S3A). We generated transgenic lines with MS2 reporter mRNA driven by enhancers from two genes active during this process: *snail* (*sna*) and *rhomboid* (*rho*). The constructs are named *sna A2.2 W0.10* and *rhoNEE*, respectively. We observed that both were expressed in patterns corresponding to their endogenous genes (Hammonds et al., 2013; Tomancak et al., 2002, 2007) (Fig. S3B): in an anterior-posterior band along the ventral side for *sna A2.2 W0.10* (Ip et al., 1992a); and in lateral stripes for *rhoNEE* (Ip et al., 1992b). At *sna A2.2 W0.10* transcription sites, we detected a progressive increase for both histone marks across cellularization (Fig. S3C,D). *rhoNEE*, on the other hand, showed no change in H3K4me1 (Fig. S3E) and an increase in H3K4me3 only between early and mid cellularization (Fig. S3F). Thus, the regulatory environments around genes can change over relatively short time scales in a manner that depends on their *cis*-regulatory regions.

### Changes in the histone environment at *rhoNEE* transcription sites over space

**Fig. 4.**
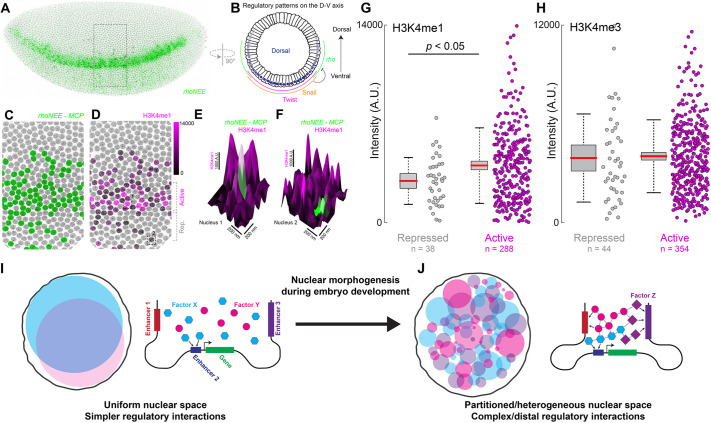
**The histone environment at *rho* transcription sites depends on their location in the embryo.** (A) The expression pattern of *rhoNEE*::MS2 mRNA on a side view of a stage 5 embryo. (B) The regulatory inputs of *rho* along the dorsal-ventral axis. Dorsal drives *rho* expression, whereas Snail represses *rho*. (C) Detailed view of the area outlined in A with nuclei transcribing *rhoNEE*::MS2 mRNA in green. (D) Same view as C, with the shade of magenta depicting the intensity of H3K4me1 at the transcription site. The active and repressed zones for *rho* expression are indicated on the right. (E,F) Detailed views of a nucleus from the active (E) and repressed (F) zone. (G,H) Quantification of (G) H3K4me1 and (H) H3K4me3 levels at *rhoNEE* transcription sites at early cellularization (see Fig. S3A). Number of transcription sites (*n*) is indicated in the figure panels. Red bar is the mean; the gray box is ±1 s.d.; the dotted lines are ±2 s.d. *P* values were calculated using a two-tailed Student's *t*-test. Only pairs with *P*<0.05 are shown. (I) The nuclear environment in early embryo development is more uniform and gene regulation may preferentially occur using fewer and more-proximal regulatory elements. (J) The space inside the nucleus undergoes ‘morphogenesis’ during embryo development, transforming it into a heterogeneous/partitioned space. Increased spatial organization during the later stages may permit complex and long-distance interactions from *cis*-regulatory regions.

After observing that histone environments at transcription sites changed over time, we investigated whether these environments also change based on the location of the cell in relationship to regulatory inputs of *rho*. The *rho* stripe (Fig. 4A, using *rhoNEE*) has a sharp ventral edge resulting from Snail repression and trails off gradually in the dorsal direction due to a decreasing gradient of the activator Dorsal (Fig. 4B) (Ip et al., 1992b). Using *rhoNEE*, we observed that, although most transcriptionally active cells are in the active region (Fig. 4C, active), some active nuclei could still be observed in the repressed region (Fig. 4C, rep.). We quantified H3K4me1 and H3K4me3 at *rhoNEE* transcription sites (Fig. 4D), sorting cells based on their location in the *rho* expression pattern (e.g. Fig. 4E,F) from stage 5 embryos at early cellularization. We observed that transcription sites in the active region had a higher level of H3K4me1 compared with sites in the repressed region (Fig. 4G). In contrast, there was no difference in H3K4me3 (Fig. 4H). Thus, the local histone environment of a gene could vary based on the time and place of its expression, potentially reflecting changes in the states of its regulatory elements.

### ‘Nuclear morphogenesis’ is integral to developmental gene regulation

During development, the embryo experiences changes in its gene regulation profiles, adapting to different regulatory needs. Early on during development, cell division occurs quickly and TFs form multi-cell gradients with uniform nuclear distributions (Fig. 4I). This stage up to gastrulation lasts ∼3 h in *D. melanogaster* embryos. Subsequently, the division slows down and segmentation of the body takes place. This phase lasts ∼18 h. During this time, many regulatory and signaling pathways for different cell types, tissues and organs become active, leading to a potentially complicated regulatory environment in the nucleus (Fig. 4J). We observed that the expression patterns of TFs became lineage specific and their nuclear distributions became heterogeneous. For TFs that regulate different genes during later stages of development, this spatial partitioning could separate them from early genes and provide another layer of safety beyond repression to prevent accidental cross-activation.

As TFs interact with DNA, their general nuclear distributions could reflect changes in the chromatin environment. Starting from the beginning of zygotic gene expression, the levels of histone modifications increase at specific regions on the genome. This occurs concurrently with the establishment of TADs and with the chromatin organizing into distinct transcriptionally active or repressed regions (Matharu and Ahituv, 2015). We observed such increases in active and repressive histone modifications. The increase in H3K27me3, associated with the polycomb group proteins (Denholtz et al., 2013), was the most drastic and could be due to differentiating cells shutting down genomic regions. As chromatin accessibility may drive sub-nuclear compartmentalization (McSwiggen et al., 2019), this change in chromatin organization could create distinct accessibility patterns that guide TFs into heterogeneous distributions. These changes in the distributions of TFs and chromatin states across development could alter the regulation of genes depending on the timing of their expression.

To quantitatively measure temporal and spatial variations in histone environments around genes, we used constructs with MS2 reporter mRNAs. We verified that their expression patterns matched their endogenous counterparts in the developmental stages we observed. Nevertheless, to make specific statements regarding the spatial-temporal behaviors, regulatory properties and functions of endogenous genes, future experiments would ideally tag endogenous genes and decouple position tracking from transcriptional activity to observe both active and repressive microenvironments.

Over time scales of hours, we observed an increase in H3K4me1 near active *hbBAC* transcription sites between stage 5 and 10 embryos. In contrast, the levels of H3K4me3 were high in both younger and older embryos. This suggests that the promotor is used at similar levels during both stages. In contrast, enhancers, especially those that are not immediately next to the promoter, may not have sufficient time to interact with the promoter in the early embryo due to the rapid division cycle and relative lack of structure in the nucleus. As the nuclear environment becomes more stable and heterogeneous in older embryos, longer-distance interactions could form, bringing more or different enhancers into play. For genes active during multiple developmental stages, a changing set of regulatory elements over time due to kinetic constraints may permit them to respond to different regulatory inputs and serve multiple roles using the same *cis*-regulator region.

In less than 1 h, during the process of cellularization, we observed that the histone environments at transcription sites of *sna A2.2 W0.10* and *rhoNEE* could still vary. The upward trend of H3K4me3 for both constructs could indicate increasing transcriptional activity in general. Although both constructs share the same *hbP2* promoter, there is still a difference in the time trajectory of H3K4me3, potentially stemming from their different enhancers. Notably, the *rhoNEE* minimal enhancer is about 300 bp while the *sna A2.2 W0.10* construct contains a longer enhancer of about 2 kb from *sna* (Perry et al., 2010). The short enhancer of *rhoNEE* available for H3K4me1 deposition could mean that small changes in H3K4me1 level are below the detection threshold, whereas *sna A2.2 W0.10* has a much more space for H3K4me1 to accumulate. Interestingly, *hbBAC*, which showed a clear change in H3K4me1, contains a *cis*-regulatory region of about 18 kb with potentially multiple enhancers (Perry et al., 2011). In addition, we observed variations in the *rhoNEE* histone environments depending on where the cell is located, consistent with the regulatory inputs of *rhoNEE*: the changing H3K4me1 but constant H3K4me3 suggests a shared active promoter (*hbP2* in our construct) but differential engagement from enhancers, depending on whether the repressor Snail is present. Together, these results imply that histone environments around genes depend on the properties and states of *cis*-regulatory elements. These differences could refine expression patterns over time, as is the case for many developmental genes (Briscoe and Small, 2015), where differences in local epigenetic marking could guide incorrectly expressing or silent cells into their proper state.

In conclusion, this survey of the distribution of TFs and histone modifications in *Drosophila* embryos has provided evidence for the emergence of a heterogeneous nucleus during development. This transformation of the nuclear space may parallel morphogenesis where the embryo body becomes increasingly complex. Thus, our work suggests that ‘nuclear morphogenesis’ is a promising hypothesis that should be further tested and refined. Through investigating the interactions between nuclear environment and gene regulation beyond gastrulation, we could gain insights into how the changing spatial architecture of the nucleus alters the strategies of gene regulation during later stages of embryo development. The ability to coordinate multiple lineage-, location- and time-specific environments in a single nucleus could facilitate gene regulation to ensure consistent cellular differentiation and the emergence of a robust developmental process. Spatial partitioning at both microscopic and macroscopic levels could be a common strategy in multi-cellular organisms to separate or coordinate regulatory processes that spans multiple length and time scales.

## MATERIALS AND METHODS

### Fly lines

All *Drosophila melanogaster* strains were maintained under standard laboratory conditions at 23°C. The line containing the *cis*-regulatory region (18 kp) of *hunchback* (*hb*) on a bacterial artificial chromosome (BAC) driving a *yellow* reporter mRNAs containing 24 MS2 stem loops generated by Bothma et al. (2015) for live imaging was used as a readout for the expression of *hb*. The reporter constructs for *rhoNEE* and *sna A2.2 W0.10* were derived from the plasmid pIB-hbP2-P2P-lacZ-αTub3 UTR (Chen et al., 2012) with the *hbP2* enhancer between the restriction sites HindIII and AscI replaced with the *rhoNEE* or the *sna A2.2 W0.10* sequence. The *hbP2* promoter was retained. The plasmids were synthesized by Genscript. The constructs were injected into Bloomington Drosophila Stock Center line 27388 and integrated using RMCE by GenetiVision. Virgin flies from a transgenic *D. melanogaster* line containing MS2 coat protein (MCP) fused to EGFP driven by the *nanos* promoter (from Garcia et al., 2013) were crossed with males from the *hbBAC*, the *rho* or the *sna A2.2 W0.10* reporter line to label transcriptions sites for further amplification using immunofluorescence (IF) staining. The MCP-EGFP line does not contain a nuclear localization signal (NLS) to minimize MCP-EGFP forming aggregates in the nucleus in the absence of MS2 mRNAs.

### Enhancer sequences

The enhancer sequences were as follows: *rhoNEE*, CTTGGGCAGGATGGAAAAATGGGAAAACATGCGGTGGGAAAAACACACATCGCGAAACATTTGGCGCAACTTGCGGAAGACAAGTGCGGCTGCAACAAAAAGTCGCGAAACGAAACTCTGGGAAGCGGAAAAAGGACACCTTGCTGTGCGGCGGGAAGCGCAAGTGGCGGGCGGAATTTCCTGATTCGCGATGCCATGAGGCACTCGCATATGTTGAGCACATGTTTTGGGGGAAATTCCCGGGCGACGGGCCAGGAATCAACGTCCTGTCCTGCGTGGGAAAAGCCCACGTCCTACCCACGCCCACTCGGTTACCTGAATTCGAGCT; and *sna A2.2 W0.10*, GTCGACCTAGTTCTGTTTTGTGACTCGGATTTACTATTTCGCATGGCTCCTCTTCGAACAATGTCAGTCGAGCTCTGTAGATCCCTGTGTTCCCTCTTCATTGTCAACTTGAACAAATGAGCCAGGGAACAAGGTGCAAAAATGGGACGGTCCTATTCTCAGCAAAAATTGACAAGAACAACAACAATGTCTATGGAAAATCGAACTTCATCCCAGCACCTGCAGAAATCCCGAGCGAGTCGGGGAAAAAGTATTTAACCCCCGAAAGGGTTTTCCCCAAAATAATGAAGTAATGAATGAAGCGGAAAACACTGGCCGCCAATCTACCTAATACTAATGAGCGGGCCAACCCGACCAGGAATTTTTGCAAGTCAGGTACTTCAACGGATATATGGGTTCGACAAGTGCGGATTTTCCCGCGACATCAATGAGGACTTGGCCGGGTTATCCGCGGTGCTCATCGGGCAATTCCGCGGCCGAGGACTTCATCGTAGTGATCATTAGGTAGATATGTGCATGGATGTGACATGGCGATCATTGCGCGGAATAACACACGTAATAACCGAGATATCCGGGATGACCCACCAGGTAGGATGTGAGGACATATAGAAAACCCCCAGCCAGTTTTTCCACTCGTCGTGGCTTGTTTTGCTTGAGTTTCGCTGACTGCGTAATTGGATAAGATGGGAAATTACTTTAAATCCTTCGCTGATCCACATCCGGACATTCGTCGAAGGAAAATCCATTGCAGGGAAATACGAAATGGAAATGCGGCTGGGTTATTGGCTCGACATTTCCCATCTTCCCTCACGCCATTGGTTGCAGGATCGCGGGGAATTGGAATTCCGCGCTGGAATTTTTTGTCACCTCTTGGGTTTATCAAAACTTTTGGGTTTGCTATGGATTTTTTCCAATTTTACCACCGCGCCTGGTTTTTTTTTTTTGACGACGCGGAAAATCGGACTTGGCTATGCGGGCTTGTCTGTTTTTCCGGGTACAAAGTCTGCATGTCAGCCTCCATGCGGGAGTGGGAGTTGGGAAAGTTTCCCATCGATAGTTGGAGGGGTGGCTTGAAAGTCTGGAGGTGCTAGCTGGGAAAGTTGTGTGTGCGCGATGAGGCAAGGAGTCAAAGATCAGGGGAGTTGGAAAGCGAGAATTGTGGGAATCGTCCAGGACTCAGCTGGATGCTGAGGGGCAGTATGATTTTTTTTACGTTATCAATCGAATTGATTTTAAGACAGCAGAACTTCACATACTAATAAGATGACCATGGGATTAGTTAAAATGTGTAACTCGTATTCGAATCGTCATTCTTTCACGGACCAATCGTGGGAACAGGAGATCTCTTCGATCCAAGCTCACAGGAGACTTGACACTCTTCGTCTATTCCTTGTCAAGTTTTTAATGACATCTCCTATGCCCTGAGCTATGTTTTCCTAGCTCTCATCGATCGCTGCCAATGAGCCACTGGAGATGATCCATAAGTCAGCGTAGAGTGCACCCCAGAGTTGACACTTGGTGTCTCGGAATTCGGCTCATTATCAGTGCTATTTTTGGAACACCTCTCTGCGAAGGTGTCATTTTTGTCAGTGCGTATCGCTCAGGTTCAACTCCCCACCAAAAACCGAATTTAGAGCATCGGCAGATGTACTTGAAGCACTCAATCTAAGTGAGGAAACCACCCCATGAACGAAGAGTACTAGGAGTCCTATTTGACTCGTGCTTAAAAATAGAAAATTACTTAGGGTGATCCATAGGTAGGGAGGCGATATTGTAACTTGCATTTCGGACCCGGACCTGCACGAGTTATTACGGGTGGGTTGTGAGCGTATCGGGAAATTGGAGAGCCACCAGATCTGTCATAACTTATACGGGGGATCCTTATTCCTGGGAGGGTGCGCCTGCGTCTGCTCTTCCGAGAGAGAGGTGGGAAATGGAGGAAGAGAGAGAGAGAGAGAGTGAGAGAGCAGGTAGAGGGAAGTGAGGGAAATACGCAATAAGGGTATGGGAAAAGTGCTGTTGTTGTTGCTAGGTAGCGACGCACACGTGCGAGTGTTTTTCTGTTTTGAAGAAGAACCACCACCAAATGG.

### Immunofluorescence staining

Embryos from *w^1118^* flies (for imaging TFs) and from crosses of the *hbBAC*, *rhoNEE* or *sna A2.2 W0.10* reporter lines with the MCP-GFP line (for imaging transcription sites and histone modifications) were collected, fixed and IF stained using previously described protocols (Crocker et al., 2015; Tsai et al., 2017). Primary antibodies were detected using secondary antibodies labeled with Alexa Fluor dyes for confocal and Airyscan imaging. Transcription sites of MS2 mRNA, Hb and Kr were imaged using Alexa488 and histone modifications were imaged with Alexa555. For STED microscopy, secondary antibodies with Alexa594 or STAR RED were used. Ubx was imaged using Alexa594, and histone modifications, Hb and En were imaged using STAR RED.

The primary antibodies and their dilution ratios were as follows: Hb (rabbit, generated for the Berkeley Drosophila Transcription Network Project (Li et al., 2008); 1:100); Kr (rabbit, generated for the Berkeley Drosophila Transcription Network Project (Li et al., 2008); 1:100); Ubx (mouse, Developmental Studies Hybridoma Bank, FP3.38-C; 1:20); En [rabbit, Santa Cruz Biotechnology (d-300), sc-28640; 1:50]; GFP [mouse, ThermoFisher, GFP monoclonal antibody (3E6), A-11120; 1:500]; H3K4me1 [rabbit, Merck, anti-monomethyl-Histone H3 (Lys4) antibody, 07-436; 1:250]; H3K4me3 [rabbit, Cell-Signaling Technology, Tri-Methyl-Histone H3 (Lys4) (C42D8) mAb, 9751; 1:250]; H3K27ac [rabbit, Active Motif, Histone H3K27ac antibody (pAb), 39133; 1:250]; H3K27me3 [rabbit, Active Motif, Histone H3K27me3 antibody (pAb), 39157; 1:250]; and H3K36me3 [rabbit, Abcam, anti-Histone H3 (tri methyl K36) antibody, ab194677; 1:250].

The secondary antibodies were as follows: Alexa488 donkey anti-mouse (Invitrogen, A21202, 1:500); Alexa488 donkey anti-rabbit (Invitrogen, A21206, 1:500); Alexa555 donkey anti-rabbit (Invitrogen, A31572, 1:500); Alexa594 donkey anti-mouse (Invitrogen, A21203, 1:500); and STAR RED goat anti-rabbit (Abberior, 2-0012-005-8, 1:250).

### Fluorescence *in situ* hybridization with post-fixation

To stain for transcription sites using RNA fluorescence *in situ* hybridization (FISH) and histone modifications using IF, we followed a previously described FISH protocol (Crocker et al., 2015; Tsai et al., 2017) and the IF protocol from the section above with the following modifications. The samples were first stained for histone modification following the IF protocol, re-fixed in 5% PFA in PBT (PBS with 0.1% Tween 20) for 20 min and then stained for *hb* following the FISH protocol. One additional primary antibody was used for the RNA FISH protocol: sheep anti-DIG (Roche, 11333089001, 1:250). One additional secondary antibody was used: Alexa488 donkey anti-sheep (Invitrogen, A11015, 1:500).

### Confocal and Airyscan microscopy

Confocal and Airyscan (Sheppard et al., 2013) imaging followed the protocols in (Tsai et al., 2017). Specifically, mounting of fixed Drosophila embryos was done in ProLong Gold+DAPI (Invitrogen, P36935). Fixed embryos at the appropriate stage and orientation were imaged on a Zeiss LSM 880 confocal microscope with FastAiryscan (Carl Zeiss Microscopy, Jena, Germany). Excitation lasers with wavelengths of 405, 488 and 561 nm were used as appropriate for the specific fluorescent dyes. Whole-embryo overviews were imaged using a Zeiss LD LCI Plan-Apochromat 25x/0.8 Imm Korr DIC M27 objective. High resolution confocal and Airyscan stacks were imaged using a Zeiss Plan-Apochromat 63x/1.4 Oil DIC M27 objective. The optimal resolution as recommended by the Zen software from Zeiss was used for the *x-y* (70.6 nm for confocal and 42.5 nm for Airyscan) and *z* direction (320 nm for confocal and 190 nm for Airyscan) of stacks used for quantification. The laser power and gain were adjusted to maximize the signal to noise ratio within the dynamic range of the PMT or Airyscan detector for each sample preparation condition (i.e. different antibodies). Within a given sample preparation condition, laser power and gain were kept the same across embryos. The acquired Airyscan stacks were processed with Zen 2.3 SP1 (Carl Zeiss Microscopy, Jena, Germany) in 3D mode to obtain super-resolved images.

### Stimulated emission depletion microscopy

Mounting of fixed *Drosophila* embryos was carried out in ProLong Diamond without DAPI (Invitrogen, P36961). Fixed embryos at the appropriate stage and orientation were imaged on a STEDYCON (Abberior Instruments, Göttingen, Germany) in 2D STED mode on an Olympus BX53 microscope. Excitation lasers with wavelengths of 594 and 640 nm were used as appropriate for the specific fluorescent dyes. The wavelength of the STED laser was 775 nm. All samples were imaged using an Olympus UPlanSApo 100× Oil/1.4 objective. The resolutions used for the *x*-*y* and *z* directions were 25 nm and 250 nm, respectively. The laser power and gain were adjusted to maximize the signal-to-noise ratio within the dynamic range of the APD detector on the STEDYCON for each sample preparation condition. Within a given sample preparation condition, laser power and gain were kept the same across embryos.

### Image processing

Image processing was carried out using Fiji (Schindelin et al., 2012) with the 3D ImageJ Suite plug-in (Schmid et al., 2010). Image processing to obtain radially averaged intensity distributions around transcription sites was carried out according to Tsai et al. (2017), with the following modification to signal normalization: for each transcription site, the maximum intensity measured in the entire radial distribution is normalized to 1, instead of normalizing to the intensity value at *r* = 0. Radially averaged auto-correlation functions were computed using an ImageJ macro (imagejdocu.tudor.lu/macro/radially_averaged_autocorrelation). Airyscan images were used for the autocorrelation of Hb and Kr for their wider field of view compared with STED, in order to include a larger number of nuclei. Intensity distributions and autocorrelation functions were plotted using Matlab (MathWorks).

## Supplementary Material

Supplementary information

Reviewer comments
