# Supplementary material for: Nuclear morphogenesis: forming a heterogeneous nucleus during embryogenesis
Source: Development. 2022 Feb 17;149(4):dev200266. doi: 10.1242/dev.200266 (PMC8918797; doi:10.1242/dev.200266)
Supplement: Supplementary information [file develop-149-200266-s1.pdf]

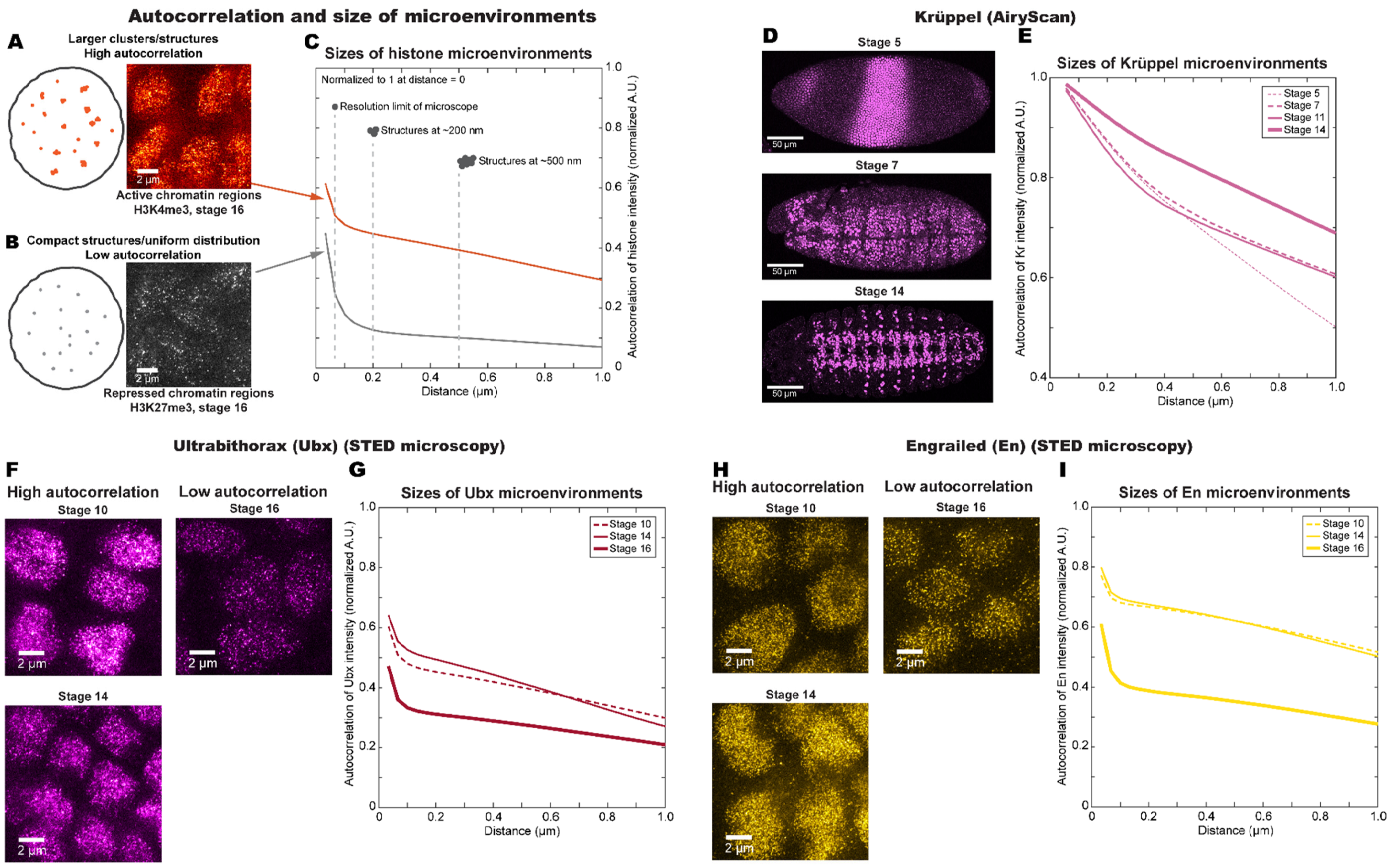
Autocorrelation and size of microenvironments
A
Larger clusters/structures
High autocorrelation
2 µm
Active chromatin regions
H3K4me3, stage 16
B
Compact structures/uniform distribution
Low autocorrelation
2 µm
Repressed chromatin regions
H3K27me3, stage 16
C
Sizes of histone microenvironments
Normalized to 1 at distance = 0
Resolution limit of microscope
Structures at ~200 nm
Structures at ~500 nm
Autocorrelation of histone intensity (normalized A.U.)
Distance (µm)
Krüppel (AiryScan)
D
Stage 5
Stage 7
Stage 14
50 µm
E
Sizes of Krüppel microenvironments
Stage 5
Stage 7
Stage 11
Stage 14
Autocorrelation of Kr intensity (normalized A.U.)
Distance (µm)
Ultrabithorax (Ubx) (STED microscopy)
F
High autocorrelation
Stage 10
Low autocorrelation
Stage 16
Stage 14
2 µm
G
Sizes of Ubx microenvironments
Stage 10
Stage 14
Stage 16
Autocorrelation of Ubx intensity (normalized A.U.)
Distance (µm)
Engrailed (En) (STED microscopy)
H
High autocorrelation
Stage 10
Low autocorrelation
Stage 16
Stage 14
2 µm
I
Sizes of En microenvironments
Stage 10
Stage 14
Stage 16
Autocorrelation of En intensity (normalized A.U.)
Distance (µm)

Fig. S1. Reading autocorrelation plots and the spatial scales of transcription factors as a function of embryo age

Related to Figure 1. (A-C) Spatial autocorrelation is a method used in geographical analysis to extract spatial features of specific sizes and quantitatively analyze spatial statistics, see, for example (Griffith, 2003), for a comprehensive treatment of the topic. In brief, the spatial autocorrelation function is calculated by taking the original image, shifting it by a given distance, and then multiplying the shifted image to the original. This product, after normalization, is the correlation of the function with itself (autocorrelation, y-axis) at that distance (x-axis). As a structure will still overlap with itself when the image is shifted by a distance comparable to its size, autocorrelation gives the relative abundance of structures/spatial features at different length scales. Panels (A-C) use histone modifications associated with open or compacted chromatin as an illustrative example (Boettiger et al., 2016; Szabo et al., 2018). (A) Transcriptionally active chromatin regions marked with H3K4me3 are relatively open, forming clusters and structures of multiple sizes that are larger than the resolution limit of the microscope (represented by a single dot in the schematic diagram). (B) Repressed chromatin regions marked with H3K27me3 are compact, with few clusters and structures larger than the resolution limit of the microscope. (C) Accordingly, the autocorrelation function of H3K4me3 shows higher correlation (y-axis) at all length scales (x-axis) versus H3K27me3. The autocorrelation plots are normalized to 1 when the distance is 0, i.e. the product of the image to an un-shifted copy. In general, the immediate drop-off in autocorrelation at the beginning of the plot reflects the resolution limit of the microscopy technique. The subsequent correlation values show the relative abundance of clusters and structures at the length scales in question. The length scales of 200 nm and 500 nm are each marked with a dotted line. (D) The distribution of another transcription factor expressed during multiple stages of embryo development Krüppel (Kr) also becomes more lineage specific as development progresses. (E) The autocorrelation function for Kr increases at longer length scales for later developmental stages. Transcription factors that are only expressed within a narrower window of embryo development, such as (F & G) Ultrabithorax (Ubx) and (H & I) Engrailed (En), show only two distinct autocorrelation states: high or low, without intermediate states as observed for Hb and Kr.

Development • Supplementary information

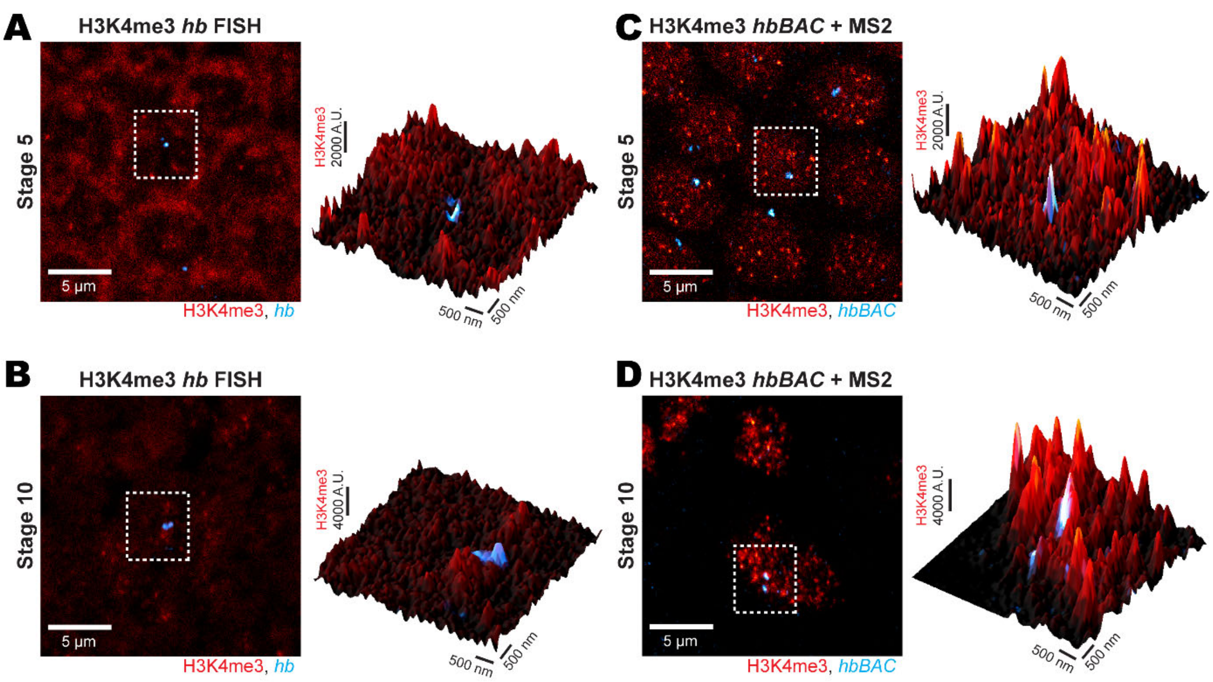

Fig. S2. Comparison of histone modification signals between transcription sites detection using FISH and MS2

Related to Figure 3. (A & B) Detection of *hb* transcription sites using fluorescence *in situ* hybridization (FISH) degrades the signal level from IF staining against histone modifications (H3K4me3 shown). (A) At stage 5, when histone modification levels are low, essentially no signal is detected for H3K4me3 inside the nucleus. (B) Only weak signals can be detected inside the nucleus at stage 10. (C & D) In contrast, IF detection of the transcription site using a MS2 reporter mRNA with MCP-GFP preserves the signals for histone modifications. The LUT intensity map is the same between A & C and between B & D.

Development • Supplementary information

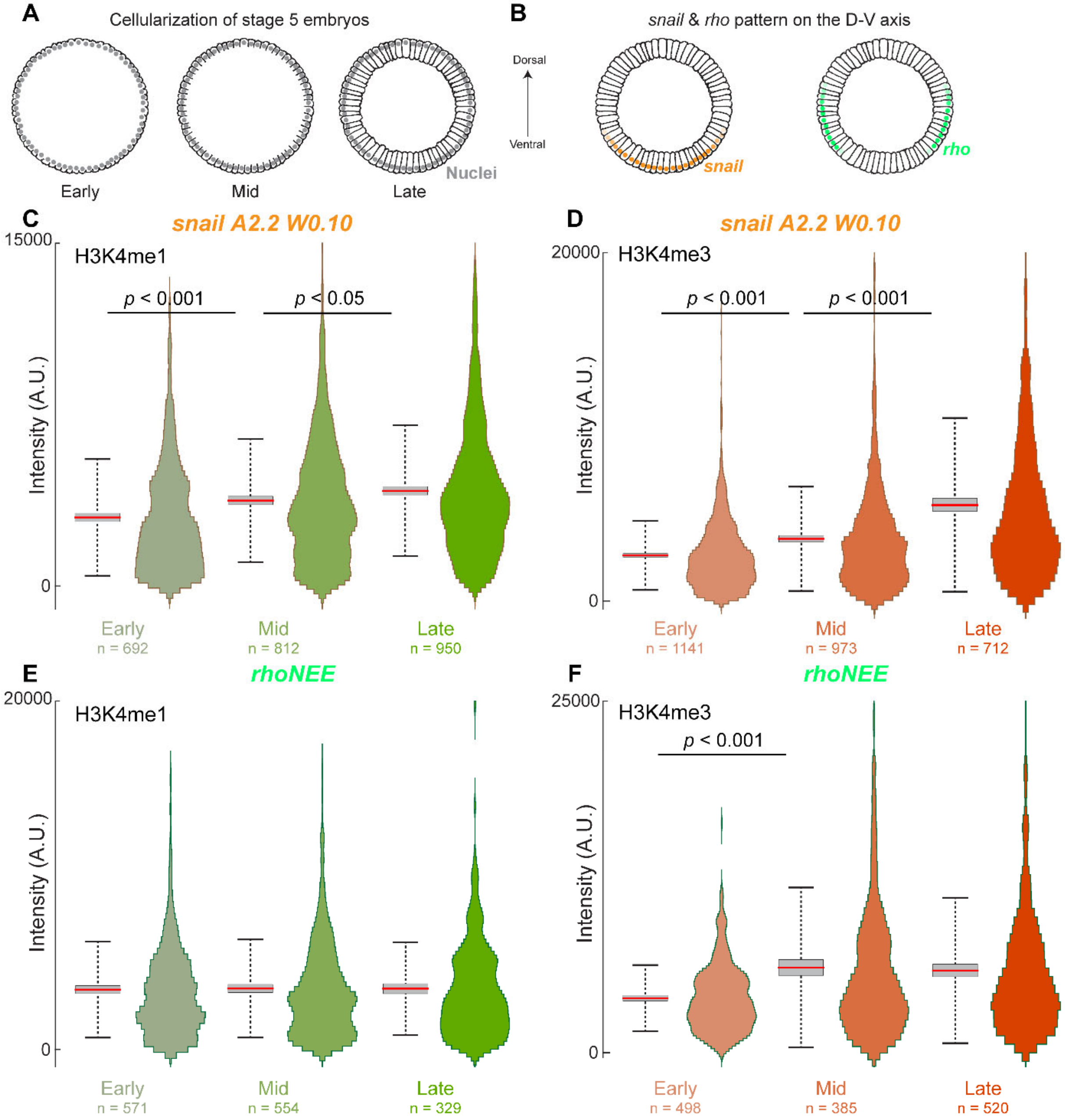

Fig. S3. Fine temporal changes in the histone environments at *snail A2.2 W0.10* and *rhoNEE* transcription sites

Related to Figure 4. (A) Stages of cellularization in stage 5 embryos, early: cell membranes begin to form, mid: cell membranes mid-way through enveloping the nuclei, and late: cellularization completed. (B) The expression patterns of the endogenous *snail* and *rho* genes along the dorsal-ventral axis, which the *snail A2.2 W0.10* and *rhoNEE* controlled transgenes recapitulate. (C-F) The distributions of H3K4me1 and H3K4me3 intensity at transcription sites of *snail A2.2 W0.10* and *rhoNEE* during cellularization. Number of transcription sites (n) is indicated in the figure panels. Red bar is the mean, the gray box is 1 s.d. and the black bars at the end of the dotted line are 2 s.d. p values from 2-tailed Student's t-test, only pairs with p<0.05 are shown.

Development • Supplementary information

Supplementary references

Boettiger, A.N., Bintu, B., Moffitt, J.R., Wang, S., Beliveau, B.J., Fudenberg, G., Imakaev, M., Mirny, L.A., Wu, C., and Zhuang, X. (2016). Super-resolution imaging reveals distinct chromatin folding for different epigenetic states. Nature *529*, 418–422.

Griffith, D.A. (2003). Spatial Autocorrelation and Spatial Filtering (Berlin, Heidelberg: Springer Berlin Heidelberg).

Szabo, Q., Jost, D., Chang, J.M., Cattoni, D.I., Papadopoulos, G.L., Bonev, B., Sexton, T., Gurgo, J., Jacquier, C., Nollmann, M., et al. (2018). TADs are 3D structural units of higher-order chromosome organization in Drosophila. Sci. Adv. *4*, eaar8082.

Development • Supplementary information
