# Supplementary material for: Nuclear morphogenesis: forming a heterogeneous nucleus during embryogenesis
Source: Development. 2022 Feb 17;149(4):dev200266. doi: 10.1242/dev.200266 (PMC8918797; doi:10.1242/dev.200266)
Supplement: Reviewer comments [file develop-149-200266_review_history.pdf]

Nuclear morphogenesis: forming a heterogeneous nucleus during embryogenesis

Albert Tsai and Justin Crocker
DOI: 10.1242/dev.200266

Editor: Benoit Bruneau

Review timeline
Original submission: 15 October 2021
Editorial decision: 16 November 2021
First revision received: 13 January 2022
Accepted: 18 January 2022

Original submission

First decision letter

MS ID#: DEVELOP/2021/200266

MS TITLE: Nuclear morphogenesis: forming a heterogeneous nucleus during embryogenesis

AUTHORS: Albert Tsai and Justin Crocker

I have now received all the referees' reports on the above manuscript, and have reached a decision. The referees' comments are appended below, or you can access them online: please go to BenchPress and click on the 'Manuscripts with Decisions' queue in the Author Area.

As you will see, the referees express considerable interest in your work, but have some significant criticisms and recommend a substantial revision of your manuscript before we can consider publication. If you are able to revise the manuscript along the lines suggested, which may involve further experiments, I will be happy receive a revised version of the manuscript. Your revised paper will be re-reviewed by one or more of the original referees, and acceptance of your manuscript will depend on your addressing satisfactorily the reviewers' major concerns. Please also note that Development will normally permit only one round of major revision.

We are aware that you may be experiencing disruption to the normal running of your lab that make experimental revisions challenging. If it would be helpful, we encourage you to contact us to discuss your revision in greater detail. Please send us a point-by-point response indicating where you are able to address concerns raised (either experimentally or by changes to the text) and where you will not be able to do so within the normal timeframe of a revision. We will then provide further guidance. Please also note that we are happy to extend revision timeframes as necessary.

Please attend to all of the reviewers' comments and ensure that you clearly highlight all changes made in the revised manuscript. Please avoid using 'Tracked changes' in Word files as these are lost in PDF conversion. I should be grateful if you would also provide a point-by-point response detailing how you have dealt with the points raised by the reviewers in the 'Response to Reviewers' box. If you do not agree with any of their criticisms or suggestions please explain clearly why this is so.

© 2022. Published by The Company of Biologists under the terms of the Creative Commons Attribution License (https://creativecommons.org/licenses/by/4.0/).

Reviewer 1

Advance summary and potential significance to field

This study by Tsai and Crocker is focused on examining the physical organization of the nucleus, which the authors state is critical for programs of gene regulation in embryogenesis. In the field there is a huge gap in understanding how exactly spatial organization of chromatin in the nucleus is tied to gene regulation. The temporal dynamics and progressive steps in nuclear reorganization and formation of heterogenous distribution of microenvironments over a range of early developmental stages at the level of individual nuclei has not been examined. This study is a positive step towards understanding the dynamics of spatial organization within the nucleus. This manuscript presents an interesting survey of temporal dynamics in the spatial organization of nuclei in early developing Drosophila embryos, using high-resolution imaging of the nucleus to monitor the location of a series of transcription factors (TFs), epigenetic histone marks and their relationship to a few select active loci.
Their findings demonstrate a change in nuclear organization of transcription factors and active enhancers as development proceeds. They show that the distributions of transcription factors, of active enhancers, and of specific histone modifications become progressively more heterogeneous in nuclear space from early to late stages. This heterogeneity is what they highlight as a key visual characteristic of reorganization of the nucleus correlated with embryogenesis and its underlying gene regulatory programs. The strength and novelty of this paper is that it provides rigorous analyses of spatial changes in nuclear organization over multiple stages to uncover general trends moving from broad distributions to increasingly heterogenous locations of TFs and epigenetic marks.

Comments for the author

This study by Tsai and Crocker is focused on examining the physical organization of the nucleus, which the authors state is critical for programs of gene regulation in embryogenesis. It is well-established at the population level that there are broad scale nuclear reorganizations in cells and tissues during progressive steps of development and differentiation. This reorganization creates heterogeneity in the nucleus that may define functionally important microenvironments. For example, these authors have previously shown (Tsai, 2017) that TFs can have asymmetric and heterogenous distributions in Drosophila nuclei and that the location of an active gene (svb) correlates with areas enriched for TFs. While existing data supports the idea of nuclear reorganization in Drosophila embryos and uncovers intriguing correlations, these analyses usually represent a snapshot of a single stage of development, generally a relatively late stage. The temporal dynamics and progressive steps in nuclear reorganization and formation of heterogenous distribution of microenvironments over a range of early developmental stages at the level of individual nuclei has not been examined.

This manuscript presents an interesting survey of temporal dynamics in the spatial organization of nuclei in early developing Drosophila embryos, using high-resolution imaging of the nucleus to monitor the location of a series of transcription factors (TFs), epigenetic histone marks and their relationship to a few select active loci. Their findings demonstrate a change in nuclear organization of transcription factors and active enhancers as development proceeds. They show that the distributions of transcription factors, of active enhancers, and of specific histone modifications become progressively more heterogeneous in nuclear space from early to late stages. This heterogeneity is what they highlight as a key visual characteristic of reorganization of the nucleus correlated with embryogenesis and its underlying gene regulatory programs.

The strength and novelty of this paper is that it provides rigorous analyses of spatial changes in nuclear organization over multiple stages to uncover general trends moving from broad distributions to increasingly heterogenous locations of TFs and epigenetic marks. It then attempts to compare the general trends with nascent transcriptional events at specific loci using transgenic reporter genes marked with MS2-MCP system. Their data on the general trends displayed by the TFs and histone marks are clear and the analyses convincing. There appears to be a systematic and progressive reorganization in the nucleus leading to spatial restrictions and heterogeneity that is highly correlated with the steps of embryogenesis. It is intriguing that results from monitoring nascent transcription of two reporter genes for aspects of hb and rho expression suggested

© 2022. Published by The Company of Biologists under the terms of the Creative Commons Attribution License (https://creativecommons.org/licenses/by/4.0/).

different correlations or preferences for histone marks compared with the general trends. The authors speculate on these differences and how they may relate to or reflect differences in the state of regulatory elements of the active genes.

A weakness of this study is that the assays on reporter genes were done using transgenes in ectopic locations under the control of a select enhancer.

Unfortunately, the MS-2 stem loops were not inserted into the endogenous hb and rho loci, containing its normal and full repertoire of regulatory elements chromatin states and interacting domains. We do not know if the preferences displayed by the transgenes accurately reflect those of the endogenous loci. The authors speculate on long-range enhancer interactions which are being inferred from enhancers in ectopic localizations within the genome. They propose that expression in later development employs interactions with distal elements hence more heterogeneity is observed at later stages in the nucleus. I do not see how their data using transgenic reporters supports this idea. These types of interpretations require support from experiments done in endogenous loci including a control gene that doesn't seem to show heterogeneity as development proceeds because it is only regulated by proximal elements. I don't see this as a fatal flaw in the study, but the authors do speculate quite a bit on the results arising from these transgenes and never mention the caveat that this may not reflect events in the endogenous loci.

In summary I feel this work is interesting and well done. It presents ideas and findings that will be of interest to the community. The paper is generally well-written and I found the references cited useful and well balanced. In the field there is a huge gap in understanding how exactly spatial organization of chromatin in the nucleus is tied to gene regulation. This study is a positive step towards understanding the spatial organization within the nucleus. Overall I would recommend for the paper for publication but encourage the authors to at least acknowledge the weakness of their MS2-MCP experiments and say that in future looking at more examples and endogenous loci will serve to test the validity of the findings.

Reviewer 2

Advance summary and potential significance to field

This study describes changes in nuclear morphogenesis in different Drosophila embryonic stages - it becoming more heterogeneous over time.

Comments for the author

This study describes changes in nuclear morphogenesis in different Drosophila embryonic stages - it becoming more heterogeneous over time. The images are convincing, and the experiment to look at the histone modifications in rho expressing cells in active vs. repressed regions is nice. But I find the paper just descriptive - I was expecting more after the 3A-H. The ideas were stated but then the paper just ended. It seems the paper is half done. There is no real advance in our understanding of transcription, chromatin, or 3D topology during embryogenesis for publication in Development. It would be better suited for a journal for descriptive studies.

Comments:
The paper is not well written. It was frustrating to read at times, and I can only say when it was confusing, and list some obvious edits to be made.
The experiment looking at histone modifications in rho-expressing nuclei is nice, however, the rationale to explain no difference in H3K4me3 between rho expressing nuclei in the active vs. repressed domains is confusing. Poised promoters are not elongating, but the assay used here is MS2 elongating signal no? It seems that this mark should be similar if both nuclei are expressing/elongating. Also, it isn't clear what insight is forthcoming from these results.
Line 45-46. Other labs (Eisen) have looked at microenvironments affecting transcription, and should be referenced.
Line 69-73. Autocorrelation analysis has to be explained in more detail. It isn't clear what this is from the text or the figure legend. Also, Fig. 1G, X-axis - from the center of what? I get the message but the approach needs to be better described.

© 2022. Published by The Company of Biologists under the terms of the Creative Commons Attribution License (https://creativecommons.org/licenses/by/4.0/).

Fig. S2 – embryos need to be bigger and the graphs smaller.
Line 129, what distal regulatory elements are you referring to here? And it isn't clear why you need to suggest that proximal regulatory elements are in use in both stages. Why wouldn't promoters be used whenever transcription occurs?
Line 133, be more specific at the end of the sentence location of the cell – you mean within the embryo?
Lne 148 – title of this section is unclear – integral part of embryogenesis for what purpose?
Line 153, 3 hours not 6 hours, no?
Line 156, why apriori is it more complicated?
Line 158, please provide more rationale here. Why couldn't repression simply prevent early genes from being activated later?
Lines 160-164. These three sentences are contradictory. Histone modifications happen before or after or concurrent with gene expression?
Line 167. We already know that changes in accessibility guide transcription factors. This sentence doesn't make sense here.
Line 173, hasn't it been shown that hb has proximal and distal enhancers that are utilized at stages 2-5? This fact seems to go against what is suggested here.
Line 176, be more specific – which enhancers of hb are used later, and where are they located compared to the early enhancers? Does the answer agree with your hypothesis? What about for other gap genes?
Edits:
Line 27, reorganizations not reorginizaitons.
Lines 58-68. Tense is odd here. Hb expression moved… should be after "We observed that…" or some other intro phrase.
Line 85, drop the from before chromatin. There are many of these grammatical errors.
Line 89 – associated seems out of place here.
Line 151, occurs not occur
Line 160, as transcription factors nor factor.

First revision

Author response to reviewers' comments

First of all, we would like to thank the reviewers for their comments, which we believe have helped us to focus our presentation with better clarity. Accordingly, we have revised the text and figures of our manuscript to address their concerns regarding potential weaknesses, explain procedures and concepts more in-depth, and make clear the implications of our findings. We would like to begin by outlining the significant changes in this revised manuscript.

1. To explore the temporal trajectories of localized regulatory environments with finer resolution, we have added a new set of experiments quantitatively measuring the trajectories of histone environments at transcription sties controlled by *rhomboid* (*rho*) and *snail* (*sna*) enhancers across cellularization during stage 5. As we sub-divided this hour-long process into three phases, we are tracking a much shorter time scale than between stages 5 and 10 that we measured for *hbBAC* (2-3 hours). Even in this short time, we detected quantitative changes in the local histone environments (H3K4me1 and H3K4me3). Interestingly, the trajectories for both histone marks of our MS2 reporter construct driven by the *rho* enhancer were different from those driven by *sna* enhancers, despite both constructs sharing a the same promoter. This suggests that the dynamics of local histone marks, even for ones associated with either the promoter or the enhancer, could in fact be a function of both. This is now the third paragraph of the section "Changing histone modifications around transcription sites over time" and Figure S3 in our revised manuscript and provides a transition into the subsequent section.
2. We have added Figure S1A-C and the corresponding figure legends to provide a primer on how spatial autocorrelation is computed and how to interpret the resulting autocorrelation plot as a metric of the abundance of structures/spatial features at different length scales. The histone marks H3K4me3 and H3K27me3 are associated with,

© 2022. Published by The Company of Biologists under the terms of the Creative Commons Attribution License (https://creativecommons.org/licenses/by/4.0/).

respectively, spatially open or compacted chromatin, which we use as an illustrative example how the presence or lack of spatial clustering affects the autocorrelation function. We also cited a comprehensive treatment of analysis using spatial autocorrelation.

3. We have added Figure S2 to provide a clear comparison between our approach of using MS2/MCP to tag transcription sites and using RNA FISH. The degraded signals from histone modifications with RNA FISH provide the motivation for our approach: clear signals from both transcription sites and histone modifications greatly simplify quantitative analysis and improve its quality.
4. We now explain that our *cis*-regulatory region::MS2 mRNA constructs recapitulated the expression patterns of their endogenous counterpart at the developmental stages we imaged. For the *hb* construct, it was previously used to track the transcription kinetics of *hb* in live imaging experiments in Bothma et al., 2015. We also now give names to our constructs distinct from the endogenous genes to avoid confusion and make clear that we are not making statements about the specific behaviors and properties of the endogenous genes. The construct names are: *hbBAC* (~18 kbp regulatory region upstream of *hb*), *rhoNEE* (~300 bp minimal enhancer from *rho*), and *sna A2.2 W0.10* (~2 kbp enhancer from *sna*). We now explicitly state in the third paragraph in the discussion (the section that begins with “Nuclear morphogenesis”…) the potential caveat of using our setup. Future experiments to specifically measure the behaviors of endogenous genes could use genome editing techniques to tag the location of genes and integrate MS2 stem-loops into their mRNAs such that their location and transcriptional state could be tracked independently. However, as this is a signification modification, finding the correct tagging approach to preserve the functions of endogenous genes would be a significant portion of the work involved.

Finally, our intention is to share, in a concise format, a preliminary yet exciting observation on how the nuclear space and transcriptional regulation interact across embryo development. Therefore, we are submitting under the shorter format of a Research Report, which our manuscript is already at the length limit. We posit that the increasing heterogeneity of transcription factors and histone marks hints at a spatial transformation in the nucleus during development analogous to the morphogenesis of the animal body. Furthermore, our measurements of how the local environments of genes change over space and time imply that said heterogeneity in a nucleus could generate multiple distinct regulatory environments, each with their own lineage-, location-, and time-dependence. Therefore, we agree that our story is still beginning; it may yet be in its first chapter. We have outlined possible paths to continue the investigation, including exploring if regulatory environments have increased importance later on during development where complex regulatory patterns are needed to guide robust cellular lineage formation and ensure correct terminal differentiation. As we seek to understand how development shapes the nuclear space and how the nuclear space in turn shapes gene regulation, there are many addition directions to continue our investigation into the spatial dynamics of developmental gene regulation.

We will now respond to specific comments from the reviewers. The reviewers’ comments will be in blue and our response in black.

Reviewer 1 Advance Summary and Potential Significance to Field:

This study by Tsai and Crocker is focused on examining the physical organization of the nucleus, which the authors state is critical for programs of gene regulation in embryogenesis. In the field there is a huge gap in understanding how exactly spatial organization of chromatin in the nucleus is tied to gene regulation. The temporal dynamics and progressive steps in nuclear reorganization and formation of heterogenous distribution of microenvironments over a range of early developmental stages at the level of individual nuclei has not been examined. This study is a positive step towards understanding the dynamics of spatial organization within the nucleus. This manuscript presents an interesting survey of temporal dynamics in the spatial organization of nuclei in early developing Drosophila embryos, using high-resolution imaging of the nucleus to monitor the location of a series of transcription factors (TFs), epigenetic histone marks and their relationship to a few select active loci. Their findings demonstrate a change in nuclear organization of transcription factors and active enhancers as development proceeds. They show that the distributions of transcription factors, of

© 2022. Published by The Company of Biologists under the terms of the Creative Commons Attribution License (https://creativecommons.org/licenses/by/4.0/).

active enhancers, and of specific histone modifications become progressively more heterogeneous in nuclear space from early to late stages. This heterogeneity is what they highlight as a key visual characteristic of reorganization of the nucleus correlated with embryogenesis and its underlying gene regulatory programs. The strength and novelty of this paper is that it provides rigorous analyses of spatial changes in nuclear organization over multiple stages to uncover general trends moving from broad distributions to increasingly heterogenous locations of TFs and epigenetic marks.

We agree with the reviewer that the relationship between spatial organization in the nucleus and gene regulation is not well established. Moreover, measuring the nuclear environment of specific cells and at specific genes in an embryos composed of populations of cells has been challenging. A technical goal of this manuscript is to demonstrate how we may use high-resolution fluorescence microscopy to perform quantitative analysis of the regulatory environments inside a single nucleus and at a single gene.

Reviewer 1 Comments for the Author:

This study by Tsai and Crocker is focused on examining the physical organization of the nucleus, which the authors state is critical for programs of gene regulation in embryogenesis. It is well-established at the population level that there are broad scale nuclear reorganizations in cells and tissues during progressive steps of development and differentiation. This reorganization creates heterogeneity in the nucleus that may define functionally important microenvironments. For example, these authors have previously shown (Tsai, 2017) that TFs can have asymmetric and heterogenous distributions in Drosophila nuclei and that the location of an active gene (svb) correlates with areas enriched for TFs. While existing data supports the idea of nuclear reorganization in Drosophila embryos and uncovers intriguing correlations, these analyses usually represent a snapshot of a single stage of development, generally a relatively late stage. The temporal dynamics and progressive steps in nuclear reorganization and formation of heterogenous distribution of microenvironments over a range of early developmental stages at the level of individual nuclei has not been examined. This manuscript presents an interesting survey of temporal dynamics in the spatial organization of nuclei in early developing Drosophila embryos, using high-resolution imaging of the nucleus to monitor the location of a series of transcription factors (TFs), epigenetic histone marks and their relationship to a few select active loci. Their findings demonstrate a change in nuclear organization of transcription factors and active enhancers as development proceeds. They show that the distributions of transcription factors, of active enhancers, and of specific histone modifications become progressively more heterogeneous in nuclear space from early to late stages. This heterogeneity is what they highlight as a key visual characteristic of reorganization of the nucleus correlated with embryogenesis and its underlying gene regulatory programs.

We are glad that the review has noted that a key question that we attempt to address in this work is a question of temporal/spatial trajectories, namely what the path is during development that the nuclear space takes to reach a heterogeneous state that we have previously observed in later stage embryos (e.g. Ubx distributions).

The strength and novelty of this paper is that it provides rigorous analyses of spatial changes in nuclear organization over multiple stages to uncover general trends moving from broad distributions to increasingly heterogenous locations of TFs and epigenetic marks. It then attempts to compare the general trends with nascent transcriptional events at specific loci using transgenic reporter genes marked with MS2-MCP system. Their data on the general trends displayed by the TFs and histone marks are clear and the analyses convincing. There appears to be a systematic and progressive reorganization in the nucleus leading to spatial restrictions and heterogeneity that is highly correlated with the steps of embryogenesis. It is intriguing that results from monitoring nascent transcription of two reporter genes for aspects of hb and rho expression, suggested different correlations or preferences for histone marks compared with the general trends. The authors speculate on these differences and how they may relate to or reflect differences in the state of regulatory elements of the active genes.

Although the visual changes in the distributions of transcription factors and histone modifications are themselves interesting, we are glad that the review has clearly noted that we attempt to

© 2022. Published by The Company of Biologists under the terms of the Creative Commons Attribution License (https://creativecommons.org/licenses/by/4.0/).

rigorously analyze, using quantitative metrics where appropriate, how spatial characteristics, such as heterogeneity and correlations between different elements, change in the nuclear environment during development.

A weakness of this study is that the assays on reporter genes were done using transgenes in ectopic locations under the control of a select enhancer. Unfortunately, the MS-2 stem loops were not inserted into the endogenous hb and rho loci, containing its normal and full repertoire of regulatory elements, chromatin states and interacting domains. We do not know if the preferences displayed by the transgenes accurately reflect those of the endogenous loci. The authors speculate on long-range enhancer interactions which are being inferred from enhancers in ectopic localizations within the genome. They propose that expression in later development employs interactions with distal elements hence more heterogeneity is observed at later stages in the nucleus. I do not see how their data using transgenic reporters supports this idea. These types of interpretations require support from experiments done in endogenous loci, including a control gene that doesn't seem to show heterogeneity as development proceeds because it is only regulated by proximal elements. I don't see this as a fatal flaw in the study, but the authors do speculate quite a bit on the results arising from these transgenes and never mention the caveat that this may not reflect events in the endogenous loci.

As we have described in item 4 on the list of significant changes, we now are careful to distinguish our reporters from their endogenous genes and include a disclaimer regarding the potential caveats. We also discuss how the different temporal/spatial behaviors of the different reporters could be explained strictly by referencing the regulatory regions that are included in the constructs. While our reporters may not completely capture the behaviors of endogenous genes, these reporters recapitulate key aspects of the expression pattern of the endogenous genes in the developmental stages that we observed (as well as the prior use of *hbBAC* for live imaging). We therefore feel that they are sufficiently representative of how genes in general could interact with the nuclear environment during development and proceed accordingly. We also now state, as the reviewer has noted, that to make definitive statements regarding the spatial temporal behaviors, regulatory properties, and functions of endogenous genes, the ideal approach would be to tag the genes themselves. However, as tagging is a significant perturbation, maintaining functionality while tagging a developmentally critical gene may require iterative optimizations and, thus, significant investments of time and effort.

In summary I feel this work is interesting and well done. It presents ideas and findings that will be of interest to the community. The paper is generally well-written and I found the references cited useful and well balanced. In the field there is a huge gap in understanding how exactly spatial organization of chromatin in the nucleus is tied to gene regulation. This study is a positive step towards understanding the spatial organization within the nucleus. Overall, I would recommend for the paper for publication but encourage the authors to at least acknowledge the weakness of their MS2-MCP experiments and say that in future looking at more examples and endogenous loci will serve to test the validity of the findings.

We would like to thank the reviewer once again for their suggestions and appreciation for the significance of applying rigorous analysis to understand how the nucleus develops with the embryo to facilitate in turn robust developmental regulation.

Reviewer 2 Advance Summary and Potential Significance to Field:

This study describes changes in nuclear morphogenesis in different Drosophila embryonic stages - it becoming more heterogeneous over time.

The reviewer's summary captures the first half of our study on the general distributions of transcription factors and histone modifications. We would like to stress that one of our contributions here is to express this change in the spatial properties of the nucleus using rigorous, quantitative metrics from physics. The second half our study views the nuclear space from the perspective of how individual genes see their local environments. An important message from the second half is that the environments that these genes experience have lineage-, temporal-, spatial-, and genetic-dependencies that need to be carefully quantified.

© 2022. Published by The Company of Biologists under the terms of the Creative Commons Attribution License (https://creativecommons.org/licenses/by/4.0/).

Reviewer 2 Comments for the Author:

This study describes changes in nuclear morphogenesis in different Drosophila embryonic stages - it becoming more heterogeneous over time. The images are convincing, and the experiment to look at the histone modifications in rho expressing cells in active vs. repressed regions is nice. But I find the paper just descriptive - I was expecting more after the 3A-H. The ideas were stated, but then the paper just ended. It seems the paper is half done. There is no real advance in our understanding of transcription, chromatin, or 3D topology during embryogenesis for publication in Development. It would be better suited for a journal for descriptive studies.

While our manuscript is descriptive, we attempt to do so in a quantitative manner. We also explain how our work sheds light on poorly understood aspects of developmental regulation: when and how do nuclear organization appear? What are the implications of a heterogeneous nuclear space on developmental gene regulation? We agree to a certain extent that our work is still preliminary and thus incomplete. However, we hope this work could be the beginning chapter of understanding the how the physical space inside the nucleus itself is an object that development shapes and how organizing this space in turn facilitates the complex regulatory needs of embryo development. We would therefore have to disagree that this manuscript is “just descriptive” in the sense that it merely describes another example what is already well studied or that it describes phenomena without quantitative measurements to explore their biological/biophysical significance.

Comments:

The paper is not well written. It was frustrating to read at times, and I can only say when it was confusing, and list some obvious edits to be made.

In our revision, we have attempted to improve the general clarity and better explain concepts that may not be clear to an interdisciplinary audience.

The experiment looking at histone modifications in rho-expressing nuclei is nice, however, the rationale to explain no difference in H3K4me3 between rho expressing nuclei in the active vs. repressed domains is confusing. Poised promoters are not elongating, but the assay used here is MS2 elongating signal, no? It seems that this mark should be similar if both nuclei are expressing/elongating. Also, it isn’t clear what insight is forthcoming from these results.

Regarding poised promoters, we agree that this was unclear and caused confusion. We mean and now state in the revision that transcription sites from both the active and repressed side are likely utilizing their active promoters (*hbP2* promoter in the construct) in a similar manner, leading to similar H3K4me3 levels. On the other hand, the presence or absence of the Snail repressor could affect the condition of the *rhoNEE* enhancer, therefore leading to differences in H3K4me1 levels. This section now follows the added contents from item number 1 from our list of changes and together they demonstrate a more general theme: regulatory environments around genes depend on space, time, cell-lineage, and regulatory elements. These tendencies could be a force (in the sense of a regulatory energy landscape) to push and refine genes into the correct expression state.

Line 45-46. Other labs (Eisen) have looked at microenvironments affecting transcription, and should be referenced.

We have already cited (Mir et al. 2017) and (Mir et el. 2018) from the Eisen Group and the Darzacq Group in the preceding paragraph. Additionally, the second question in our original manuscript “How are these microenvironments affecting transcriptional regulation?” may be the source of the reviewer’s question. The question is now “How and when do genes interact with these microenvironments?” to better clarify our intentions. We believe that while the citations we provided in the preceding paragraph worked toward this understanding, the question itself remains unresolved.

Line 69-73. Autocorrelation analysis has to be explained in more detail. It isn’t clear what this is from the text or the figure legend. Also, Fig. 1G, X-axis - from the center of what? I get the message but the approach needs to be better described.

© 2022. Published by The Company of Biologists under the terms of the Creative Commons Attribution License (https://creativecommons.org/licenses/by/4.0/).

We agree that autocorrelation was not explained well for a diverse audience. We now explain autocorrelation in more detail in Figure S1A-C and the accompanying figure legends, including a citation for a comprehensive treatment of using spatial autocorrelation to extract spatial features and conduct spatial statistics. Also, the x-axis is now just “Distance”, which is a standard way to label the x-axis for autocorrelation plots.

Fig. S2 – embryos need to be bigger and the graphs smaller.

For Figure S1, we have expanded the images of the embryos and nuclei and they now occupy roughly the same amount of space as the quantitative plots, which are a central aspect of our presentation. There was no Figure S2 in the original manuscript.

Line 129, what distal regulatory elements are you referring to here? And it isn’t clear why you need to suggest that proximal regulatory elements are in use in both stages. Why wouldn’t promoters be used whenever transcription occurs?

We have now clarified this statement where proximal regulatory elements = promoters and more distal elements = enhancers, which we now realize is not a commonly held definition. The sentence is now in the fourth paragraph of the discussion and we state regarding the promoter: “This suggests that the promotor is utilized at similar levels during both stages.” We also now describe in the same paragraph how the usage of enhancers may differ between stages.

Line 133, be more specific at the end of the sentence location of the cell – you mean within the embryo?

This sentence now ends with: “…the location of the cell in relationship to regulatory inputs of *rho*.”

Lne 148 – title of this section is unclear – integral part of embryogenesis for what purpose?

This is the final discussion section for the shorter report format, and the new title is now “Nuclear morphogenesis is integral to developmental gene regulation”. We mean that forming the nuclear space during development is an important process in facilitating the complex interactions necessary for developmental gene regulation.

Line 153, 3 hours not 6 hours, no?

We corrected this mistake; embryo development up to gastrulation takes about 3 hours.

Line 156, why apriori is it more complicated?

We did not state that the environment is “more complicated”, only that it is “complicated”, with the rationale given in part of the sentence immediately preceding it.

Line 158, please provide more rationale here. Why couldn’t repression simply prevent early genes from being activated later?

We are not stating that physical separation occurs to the exclusion of repression. We would simply like to highlight another possibility. To clarify this, the sentence now reads: “For TFs that regulate different genes during later stages of development, this spatial partitioning could separate them from early genes and provide another layer of safety beyond repression to prevent accidental cross- activation.”

Lines 160-164. These three sentences are contradictory. Histone modifications happen before or after or concurrent with gene expression?

We are not sure what the review means here as we have made no statement trying to assign a specific temporal ordering. The first sentence is a statement that as TFs interact with the DNA, changes in the chromatin environment may also alter the localization/clustering of TFs. The next two sentences summarize previous findings: transition to zygotic gene expression marks the

© 2022. Published by The Company of Biologists under the terms of the Creative Commons Attribution License (https://creativecommons.org/licenses/by/4.0/).

beginning of clear increases in many histone marks (e.g. H3K4me1, H3K4me3, and H3K27me3) and chromatin organization in the form of TADs also appear around this time. Of the three options the reviewer listed, all are possible and not mutually exclusive.

Line 167. We already know that changes in accessibility guide transcription factors. This sentence doesn't make sense here.

There is indeed evidence that chromatin accessibility guides TFs, which supports our claim that changes in the heterogeneity of transcription factor distributions that of histone modifications are related. To explicitly state this, the sentence now reads: "As chromatin accessibility may drive sub- nuclear compartmentalization (McSwiggen et al., 2019), this change in chromatin organization could create distinct accessibility patterns that guide TFs into heterogeneous distributions."

Line 173, hasn't it been shown that hb has proximal and distal enhancers that are utilized at stages 2-5? This fact seems to go against what is suggested here.

As noted in our response to the comment below, we have revised the paragraph and we are no longer making any claims of which specific enhancers are in use. We meant to use proximal regulatory elements as a generic term for promoters, which may be a part of the confusion. Regarding *hb* shadow and primary enhancers being utilized before zygotic transition in stage 5, we are not sure about the specific work the reviewer is referring to.

Line 176, be more specific – which enhancers of hb are used later, and where are they located compared to the early enhancers? Does the answer agree with your hypothesis? What about for other gap genes?

As the *hbBAC* construct contains a ~18 kbps region upstream of *hb*, potentially containing multiple enhancers, we are not able to pinpoint a specific enhancer that is in use. We also are not making specific statements of how and when specific enhancers of *hb* are engaged, or other gap genes for that matter. We are trying to make a general argument from kinetics: the more stable nuclear environments during later stages of development may permit more time for interactions, increasing the chances that more enhancers, especially beyond the most proximal ones, come into play. We have revised this part of our discussion to clarify our reasoning:

> Over time scales of hours, we observed an increase in H3K4me1 near active hbBAC transcription sites between stage 5 and 10 embryos. In contrast, the levels of H3K4me3 were high in both younger and older embryos. This suggests that the promotor is utilized at similar levels during both stages. In contrast, enhancers, especially ones not immediately next to the promoter, may not have sufficient time to interact with the promoter in the early embryo due to the rapid division cycle and relative lack of structure in the nucleus. As the nuclear environment becomes more stable and heterogeneous in older embryos, longer-distance interactions could form, bringing more or different enhancers into play. For genes active during multiple developmental stages, a changing set of regulatory elements over time due to kinetic constraints may permit them to respond to different regulatory inputs and serve multiple roles using the same cis- regulator region.

Edits:

Line 27, reorganizations not reorginizaitons.

Fixed.

Lines 58-68. Tense is odd here. Hb expression moved… should be after "We observed that…" or some other intro phrase.

We now have our experimental observations described using the past tense. We begin with "We observed that…" for stage 5 and continue from there.

Line 85, drop the from before chromatin. There are many of these grammatical errors.

© 2022. Published by The Company of Biologists under the terms of the Creative Commons Attribution License (https://creativecommons.org/licenses/by/4.0/).

Our sentence reads: "Because TFs interact with the chromatin…". There is no "from" in our sentence.

Line 89 – associated seems out of place here.

The sentence was missing a phrase, it is now corrected to read: "We therefore imaged histone modifications associated with different chromatin features using high-resolution confocal microscopy…"

Line 151, occurs not occur

Fixed.

Line 160, as transcription factors nor factor.

Fixed.

Second decision letter

MS ID#: DEVELOP/2021/200266

MS TITLE: Nuclear morphogenesis: forming a heterogeneous nucleus during embryogenesis

AUTHORS: Albert Tsai and Justin Crocker
ARTICLE TYPE: Research Report

I am happy to tell you that your manuscript has been accepted for publication in Development, pending our standard ethics checks.

Reviewer 1

Advance summary and potential significance to field

The authors have made extensive revisions to their original manuscript which have significantly improved the study. The original version was high quality and represented an important advance. However, the authors have added new experiments clarified analyses, and modified several discussion points which address the reviewers points in an effective manner and enhance the overall impact of the work. This helps to advance the field and is worthy of publication in Development.

Comments for the author

The authors have made extensive revisions to their original manuscript which have significantly improved the study. The original version was high quality and represented an important advance. However, the authors have added new experiments clarified analyses, and modified several discussion points which address the reviewers points in an effective manner and enhance the overall impact of the work. This helps to advance the field and is worthy of publication in Development.

It is nice to see authors make such a conscientious effort in their revisions and this increases its value and impact for general readers.

© 2022. Published by The Company of Biologists under the terms of the Creative Commons Attribution License (https://creativecommons.org/licenses/by/4.0/).
